# Deep carious lesions and their management among Finnish adolescents: a retrospective radiographic study

**DOI:** 10.1007/s00784-022-04599-3

**Published:** 2022-07-04

**Authors:** Katri Croft, Sari Kervanto-Seppälä, Eero Kerosuo

**Affiliations:** 1 Department of Oral and Maxillofacial Diseases, University of Helsinki, PO Box 41 (Haartmaninkatu 1), 00014 Helsinki, Finland; 2 Vantaa Health Center, Vantaa, Finland

**Keywords:** Deep carious lesions, Prevalence, Bitewing radiograph, Intraoral radiograph, Epidemiology

## Abstract

**Objective:**

The objective of this retrospective study was to find out (i) the prevalence of deep carious lesions, both untreated and previously treated, among 14- and 15-year olds and (ii) how deep carious lesions were managed in a Finnish public health care setting.

**Materials and methods:**

A random sample of 278 patients was taken from 3990 patients at the oral health care of the City of Helsinki. Radiographic subsample consisted of patients with bitewing and periapical radiographs (*n* = 128, 46% of the total sample). Deep carious lesions (extending to at least the inner half of dentine), deep restorations, direct pulp cappings, root canal treatments, and extractions in permanent premolars and molars were recorded from the radiographs. Patients with untreated deep carious lesions were followed up for 24 months.

**Results:**

In the total sample 12% had at least one untreated deep carious lesion, 10% at least one deep restoration, and 19% at least one untreated or previously treated deep carious lesion. The follow-up cohort included 48 deep carious lesions in 26 patients. Complete excavation was the most frequently chosen method (81% for lesions reaching the inner half of dentine and 56% the inner third or deeper), followed by stepwise excavation (19% and 37%, respectively).

**Conclusions:**

One-fifth of 14–15-year-olds had at least one untreated or previously treated deep carious lesion. The choice for the carious tissue removal did not follow the current recommendations for less invasive methods.

**Clinical relevance:**

Continuing education is needed to improve the diagnostics and management of deep carious lesions.

## Introduction

Despite the significant decline in the prevalence of dental caries in Western countries in the latter half of the twentieth century, deep carious lesions remain a challenge to the dental profession. The definition of a deep carious lesion varies in literature including lesions extending to at least the inner half of dentine [1], the inner third [2] or inner quarter [3, 4]. According to previous studies in Nordic countries, 22–26% of young adults had at least one untreated or previously treated deep carious lesion [1, 3]. These studies did not include Finland, hence the need for this prevalence study.

Traditionally dentists have aimed at complete or nonselective excavation to hard dentine before placing a restoration [5, 6]. When managing a deep carious lesion, this approach often leads to a pulpal exposure [7]. Alternative management strategies for deep carious lesions have been suggested to preserve the vitality of the pulp: stepwise excavation or selective excavation to soft dentine [7–10]. The results in maintaining the vitality of the pulp have been significantly better for selective excavation completed in one visit, compared with stepwise excavation requiring two visits [10, 11].

A recent systematic review concluded that both stepwise excavation and selective excavation to soft dentine reduced the risk of pulpal exposure but the superiority of either of the techniques could not be determined based on the available evidence [12]. The present International Caries Consensus Collaboration guidelines recommend choosing either of these techniques for a deep carious lesion extending to at least the inner third of dentine [2]. This approach, however, has recently been challenged in the position statement of the European Society of Endodontology (ESE). The ESE position statement recommends complete excavation under magnification and strict aseptic protocol for deep carious lesions extending to the inner quarter of dentine, if a radio-opaque zone of sound dentine cannot be detected between the lesion and the pulp [4]. There is therefore a conflict in the recommendations and a call for further research in the field.

Furthermore, questionnaire studies have shown no uniform method for managing deep carious lesions. For example, complete excavation was preferred by the majority of dentists in the USA, Brazil, and France, whereas less invasive excavation strategies, especially stepwise excavation, were preferred in Norway and Finland [13–16].

The objectives of this study were (i) to find out the prevalence of untreated and previously treated deep carious lesions among 14- to 15-year-old adolescents in Finland and (ii) to find out how deep carious lesions were managed in a Finnish public health care setting.

## Materials and methods

Up to the age of 18 years, all Finnish children are entitled to free-of-charge oral health care by the publicly funded municipality dental clinics. Dental examination intervals are mostly based on individual risk assessment. However, certain age groups receive automatically an invitation for a dental examination.

**Fig. 1:**
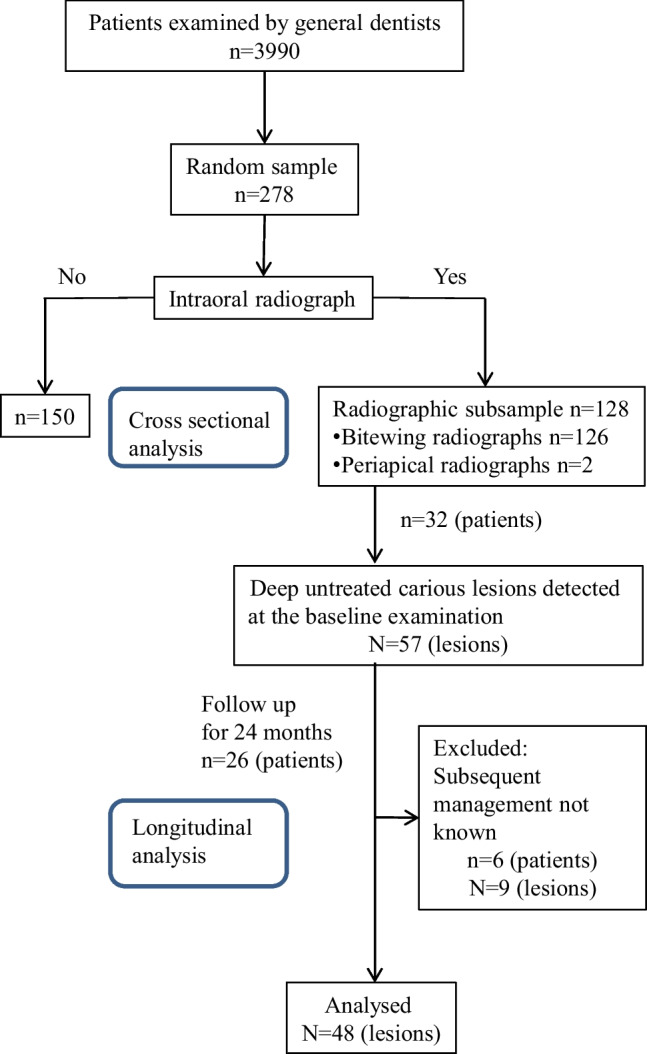
Flow chart of the study design

This study was conducted among 14- to 15-year-old pupils (8^th^ graders), all of whom are invited for a dental examination by the oral health care of the City of Helsinki. The target population consisted of the inhabitants of Helsinki born in year 2003 during the years 2017–2018 (*n* = 5280 in 2017) [17]. The source population consisted of 3990 patients from the target population, examined by general dentists at the oral health care of the City of Helsinki during the observation period of 2017–2018. The study design is presented at a separate flow chart (Fig. 1).

Based on previous studies in Nordic countries, the prevalence of deep carious lesions and deep restorations was estimated to be about 20%. Power (1-beta) was defined at 0.95 and confidence interval at 95%. The optimal number of patients (246) was assessed by the epidemiologic calculation tool EpiTools [18]. To overcome possible incomplete or missing data, additional 20% was included, adding up to a random sample of 278 patients.

Data collection took place between July 2020 and February 2021. Patients with bitewing (*n* = 126) or periapical (*n* = 2) radiographs taken during the observation period comprised a radiographic subsample (*n* = 128), covering 46% of the original sample. The digital radiographs were analyzed by the main researcher (K.C.) in a recommended dim room using the magnification, brightness, and contrast tools of the Digora Optime software (Soredex, Helsinki, Finland). The findings in occlusal and approximal surfaces in permanent premolars, as well as first and second molars, were recorded. Teeth not adequately visible in radiographs were excluded.

In this study, a lesion was considered to be deep if it extended to at least the inner half of dentine. Deep lesions were recorded in four categories: lesions extending to the inner (i) half, (ii) third, (iii) quarter of dentine, or (iv) into the pulp. Deep restorations were also recorded extending to the inner half, third, or quarter of dentine. Direct pulp cappings, partial or coronal pulpotomies, root canal treatments, and extractions due to caries were also recorded. Digital dental records were also available and used to exclude carious lesions or restorations on buccal or lingual surfaces. Previous dental records were used to verify previously performed direct pulp cappings and the reasons for extractions.

Background factors were recorded based on digital dental records: gender, self-reported general health, dental anxiety, DMFT and DMFS indices, caries risk assessment, recall interval, caries control measures, diet counselling, and self-reported dental hygiene. Apart from gender, these factors were defined by general dentists who carried out the dental examination and performed the treatment procedures. Caries control measures were performed both by general dentists and dental hygienists.

This retrospective study was mostly cross-sectional apart from one identified cohort which was also followed up longitudinally. There were originally 32 patients diagnosed with 57 untreated deep carious lesions, extending to at least the inner half of dentine at the time of the dental examination. Out of these cases, 9 teeth were excluded as there was no management information available, thus leaving a final follow-up cohort of 26 patients with 48 deep carious lesions. These cases were followed up for 24 months to find out how deep carious lesions were managed and caries control measures practiced.

Radiological calibration with a senior radiologist took place before data collection. Intra- and inter-observer reproducibility were assessed in two stages separately by the main researcher and the senior radiologist. For the first stage, 30 bitewing radiographs were randomly selected from the sample and analyzed. Teeth with a deep carious lesion extending to at least the inner half of dentine were recorded. The second stage included 30 bitewing radiographs that were preselected from the sample, including only radiographs with deep carious lesions extending to at least the inner half of dentine. The depth of each carious lesion was recorded. The radiographs were re-examined after 2 weeks by the main researcher for the intra-observer reproducibility testing.

Statistical analyses were performed using SPSS software version 27.0.1.0. The reproducibility of the radiographic interpretation was assessed by kappa statistics. For the statistical analyses, the outcomes were defined as having at least (i) one untreated deep carious lesion or (ii) one untreated or previously treated deep carious lesion. The cutting point was set at the inner half, third or quarter of dentine. Cross-tabulation tests were performed to study the association between background factors and the outcome.

Cross-tabulation tests were followed by binary multivariable regression analyses to study the background factors and their association with the outcome. For these analyses, the outcomes were defined as (i) having at least one untreated deep carious lesion, extending to at least the inner half of dentine and (ii) having at least one untreated or previously treated deep carious lesion, extending to the inner quarter of dentine. Only factors with a significant *p*-value (*p* < 0.05) in the cross-tabulation tests were included in the regression analysis. Pearson chi-square test was the primary test used, but when the nature of the data did not allow the use of Pearson chi-square test, Fisher’s exact test was used instead. Odds ratios (OR) and confidence intervals (CI) were calculated in the logistic regression analysis. Level of significance was set at *p* < 0.05 and 95% CI. The Hosmer–Lemeshow test was used to study the goodness of fit of the models.

Research permission including an ethical assessment was granted by the City of Helsinki 18^th^ May 2020. A separate review by an ethical committee was not required for a register study according to the Finnish legislation. All data was pseudonymized for the analysis and personal IDs or other identifiers were removed.

## Results

The digital patient records and digital radiographs of the sample (*n* = 278) were studied and analyzed. Detailed analysis was performed only for the radiographic subsample (*n* = 128). The gender ratio was similar in the study sample and in the target population (*p* = 0.650). The mean DMFT value was 1.81 (range 0–24) in the source population, 1.62 (range 0–12) in the total sample and 2.49 (range 0–12) in the radiographic subsample.

Radiographs were taken from 46% of the study sample. In the source population, bitewing radiographs were similarly taken from only 51% of subjects. The vast majority (69%) of patients without radiographs had no caries experience (DMFT value 0; initial lesions not taken into consideration), while 25% had DMFT value 1–3 and 7% had DMFT value 4–9. Radiographs were taken significantly more often among those with a high assessed caries risk (*p* < 0.001) and a recall interval of less than 18 months (*p* < 0.001).

At the first stage of reproducibility testing, the intra-observer reproducibility had a kappa value of 0.89. This calculation was based on a 2 × 2 table (carious lesion extending to at least the inner half of dentine vs. no deep carious lesion). At the second stage, the intra-observer reproducibility had a kappa value of 0.71. At this stage, the calculation was based on a 4 × 4 table (including carious lesions extending to the inner half/third/quarter of dentine or into the pulp). The inter-observer reproducibility had a kappa value of 0.52 for both stages.

A total of 1801 premolars and molars were included in the analysis. A total of 114 teeth (6% of teeth) had an untreated or previously treated deep carious lesion extending to at least the inner half of dentine (Table 1). Deep carious lesions (*n* = 57) and deep restorations (*n* = 46) were the most common findings, whereas direct pulp cappings (*n* = 4), root canal treatments (*n* = 3), and extractions due to caries (*n* = 4) were rare.

**Table 1:** Number of teeth with untreated or previously treated deep carious lesions in teeth visible in intraoral radiographs. *N* = 1801

	Deep carious lesion *n* (%)	Deep restoration *n* (%)	Direct pulp capping *n* (%)	Root canal treatment *n* (%)	Extraction *n* (%)	Subtotal *n* (%)	No involvement *n* (%)	Grand total *n* (%)
Depth in dentine^a^								
Inner half	25 (1.4)	20 (1.1)						
Inner third	15 (0.8)	10 (0.6)						
Inner quarter	17 (0.9)	16 (0.9)						
Total	57 (3.2)	46 (2.6)	4 (0.2)	3 (0.2)	4 (0.2)	114 (6.3)	1687 (93.7)	1801 (100.0)

^a^Applies to lesions and restorations only

In the radiographic subsample, 25% of patients had at least one untreated deep carious lesion and 21% had at least one restoration extending to at least the inner half of dentine (Table 2). When untreated and previously treated deep carious lesions were combined, 42% had at least one tooth involved. In the total sample including also patients without radiographs, 12% had at least one untreated deep carious lesion, 10% had at least one deep restoration, and 19% at least one untreated or previously treated deep carious lesion, all of these extending to at least the inner half of dentine (Table 2). The number of deep carious lesions per patient ranged between 1 and 8: 14% had one lesion, 7% had two lesions, and 4% had three or more lesions (Table 3).

**Table 2:** Number of patients with at least one deep carious lesion (untreated or previously treated). Total sample, *N* = 278. Subsample of patients with radiographs, *N* = 128

	At least one untreated deep carious lesion			At least one deep restoration			At least one deep carious lesion, untreated and previously treated^a^ combined		
	*n*	% of total sample (cumulative %)	% of subsample (cumulative %)	*n*	% of total sample (cumulative %)	% of subsample (cumulative %)	*n*	% of total sample (cumulative %)	% of subsample (cumulative %)
Depth in dentine									
The inner half	16	5.8 (5.8)	12.5 (12.5)	9	3.2 (3.2)	7.0 (7.0)	20	7.2 (7.2)	15.6 (15.6)
The inner third	5	1.8 (7.6)	3.9 (16.4)	5	1.8 (5.0)	3.9 (10.9)	7	2.5 (9.7)	5.5 (21.1)
The inner quarter	11	4.0 (11.5)	8.6 (25.0)	13	4.7 (9.7)	10.2 (21.1)	27	9.7 (19.4)	21.1 (42.2)
No involvement	96	34.5 (46.0)	75.0 (100.0)	101	36.3 (46.0)	78.9 (100.0)	74	26.6 (46.0)	57.8 (100.0)
Subtotal	128	46.0 (46.0)	100.0 (100.0)	128	46.0 (46.0)	100.0 (100.0)	128	46.0 (46.0)	100.0 (100.0)
No radiographs	150	54.0 (100.0)		150	54.0 (100.0)		150	54.0 (100.0)	
Grand total	278	100.0 (100.0)		278	100.0 (100.0)		278	100.0 (100.0)	

^a^Includes restorations, direct pulp cappings, root canal treatments, and extractions

**Table 3:** Number of teeth per patient with deep carious lesions (untreated or previously treated). Lesion depth to at least the inner half of dentine. Subsample of patients with radiographs, *N* = 128

	Untreated			Previously treated^a^			Untreated or previously treated		
*n* of teeth per pt	*n*	%	Cumulative %	*n*	%	Cumulative %	*n*	%	Cumulative %
1	18	14.1	14.1	14	10.9	5.0	23	18.0	18.0
2	9	7.0	21.1	6	4.7	15.6	16	12.5	30.5
3	3	2.3	23.4	5	3.9	19.5	8	6.3	36.7
4	1	0.8	24.2	4	3.1	22.7	4	3.1	39.8
5							1	0.8	40.6
6							1	0.8	41.4
8	1	0.8	25.0				1	0.8	42.2
No involvement	96	75.0	100.0	99	77.3	100.0	74	57.8	100.0
Total	128	100.0	100.0	128	100.0	100.0	128	100.0	100.0

^a^Includes restorations, direct pulp cappings, root canal treatments, and extractions

The association between the extent of caries control measures registered by dental professionals and at least one deep carious lesion to the inner half or quarter of dentine was studied but no significant association was found (*p* = 0.101 and *p* = 0.110, respectively). There was an association between comprehensive caries control measures and high caries risk category (*p* = 0.003), although none or only small-scale caries control measures were registered for one third of high caries risk patients.

According to the multivariable logistic regression analysis, an increase of one unit in the DMFT value resulted in a significantly increased risk of having at least one deep carious lesion to the inner half of dentine (*OR* = 1.3, CI 1.1–1.6). The risk of having at least one untreated or previously treated deep carious lesion to the inner quarter of dentine was significantly higher among female patients (*OR* = 4.6, CI 1.5–14.0) and those in high caries risk category (*OR* = 4.4, CI 1.3–14.4) (Table 4).

**Table 4:** The influence of the background factors on the outcome. Binary multivariable logistic regression analysis. Subsample of patients with radiographs, *N* = 128

	At least one untreated deep carious lesion, depth > inner half of dentine		At least one untreated or previously treated deep carious lesion, depth > inner quarter of dentine	
*Background variable*	OR^a^ (95% CI^b^)	*p*-value	OR (95% CI)	*p*-value
High caries risk category (vs. other risk categories)	1.1 (0.4–3.1)	0.883, N.S.^c^	4.4 (1.3–14.4)	0.015
Planned RC interval < 18 months (vs. ≥ 18 months)	1.9 (0.6–5.9)	0.267, N.S	2.0 (0.6–6.5)	0.263, N.S
DMFT index (continuous)	1.3 (1.1–1.6)	0.003	N.T.^d^	-
Dental anxiety reported (vs. not reported)	N.T	-	3.4 (0.4–29.5)	0.272, N.S
Diet counseling given (vs. not given)	N.T	-	2.2 (0.8–6.3)	0.140, N.S
Gender female (vs. male)	N.T	-	4.6 (1.5–14.0)	0.007
H-L^e^	0.584		0.869	

^a^*OR*, odds ratio

^b^*CI*, confidence interval

c*N.S*., non-significant

d*N.T*., not tested

e*H-L*, Hosmer–Lemeshow test for goodness of fit

The follow-up cohort included 48 deep carious lesions in 26 patients. Complete excavation was the most frequently chosen method regardless of the lesion depth (81% for lesions reaching the inner half of dentine and 56% for the inner third or deeper) (Table 5). Stepwise excavation was chosen for 19% of lesions reaching the inner half of dentine and for 37% of lesions in the inner third of dentine or deeper. Two teeth, both with a deep carious lesion extending to the pulp, were extracted; one during treatment under general anesthesia and the other as a result of acute pain and poor restorability. The difference between routinely chosen complete excavation and other management methods (stepwise excavation or extraction) was not statistically significant when the threshold of lesion depth was set at the inner third of dentine (*p* = 0.064), but became statistically significant among lesions reaching the inner quarter of dentine (*p* = 0.008) (Table 5). No root canal treatments were primarily planned, but two teeth were endodontically treated after occurrence of acute pain following either complete or stepwise excavation. Before the management decision, only two periapical radiographs were taken and only one sensibility test was performed (in 4% and 2% of cases, respectively). Follow-up radiographs were taken from five teeth (11%) after complete or stepwise excavation. In addition, sensibility tests were performed for four teeth (9%) during the follow-up. During the 24-month follow-up period, only 10 patients (39%) had new bitewing radiographs taken and four patients (15%) developed at least one new deep carious lesion.

**Table 5:** The primary management decision for each deep carious lesion according to the depth category. *N* = 48

Depth in dentine	Management decision			
	Complete excavation *n* (%)	Stepwise excavation *n* (%)	Extraction *n* (%)	Total *n* (%)
Inner half	17 (81.0)	4 (19.0)	0 (0.0)	21 (100.0)
Inner third	9 (75.0)	3 (25.0)	0 (0.0)	12 (100.0)
Inner quarter	6 (50.0)	6 (50.0)	0 (0.0)	12 (100.0)
Into the pulp	0 (0.0)	1 (33.3)	2 (66.7)	3 (100.0)
Total	32 (66.7)	14 (29.2)	2 (4.2)	48 (100.0)

Complete excavation vs. other methods/lesion reaching inner third vs. other (2 × 2 table): *p* = 0.064, N.S

Complete excavation vs. other methods/lesion reaching inner quarter vs. other (2 × 2 table): *p* = 0.008

## Discussion

The source population of the present study (3990 patients examined between 1.1.2017 and 31.12.2018) covered 76% of the target population. The gender ratio did not differ significantly between the study sample and the target population. Also the mean DMFT value (1.62) of the study sample corresponded well with the mean DMFT value (1.81) in the source population. Thus, it can be assumed the random sample is representative of the age group in Helsinki. For nationwide comparison, the mean DMFT values were compared to the corresponding values in the City of Oulu in Northern Finland among 14-year-olds in 2017–2018. The mean DMFT value in Oulu was 1.93 (range 0–25) (Chief Dental Officer of the City of Oulu, personal communication), which was only slightly higher than the mean DMFT value 1.81 (range 0–24) in Helsinki. Therefore, with some caution, the results can be used to draw conclusions about the prevalence of untreated and previously treated deep carious lesions in Finland.

The retrospective study design has got limitations. The data regarding the patients’ clinical status and background factors was not systematically recorded by the dentists. Also, the percentage of bitewing radiographs was surprisingly low in the sample (46%), compared to studies in other Nordic countries, where radiographs were available for 94% and 95% of the sample [1, 3]. Our results are very likely to underestimate the prevalence of untreated and previously treated deep carious lesions, as 25% of those with no radiographs had DMFT value 1–3 and 7% had DMFT value 4–9. It is reasonable to assume that there were untreated and previously treated deep carious lesions among these patients which were not part of the radiological subsample; thus, these lesions were unrecorded.

The intra-observer reproducibility was considered almost perfect (stage one) and substantial (stage two). The inter-observer reproducibility was considered moderate [19]. The value of bitewing radiographs in detecting especially approximal and also occlusal carious lesions has been shown in previous studies [20, 21]. This applies also to low-caries populations like our study population [22]. The Finnish national guidelines recommend taking bitewing radiographs if at least one carious lesion extending into dentine is diagnosed at the clinical examination [23]. Bitewing radiographs are also recommended if the patient has several active enamel lesions or risk factors for caries or if hidden carious lesions are suspected. According to the patient records there were several patients with carious lesions extending to dentine but no radiographs taken, which is in contrast with the national guidelines. In addition, the suboptimal positioning and projection lowered the diagnostic value of several radiographs. This is in line with a recent study showing the compromised quality of almost half of bitewing radiographs [24]. Continuing education is needed to improve the level of diagnostics, by guiding dentists to take technically optimal bitewing radiographs according to the agreed clinical guidelines.

The prevalence of deep carious lesions or deep restorations has not been studied in Finland before. In our data, the prevalence of at least one deep restoration extending to at least the inner half of the dentine was 21% for patients with radiographs. The corresponding figure in a similar study among 15-year-old adolescents in Southern Sweden was 22% [1], which falls well in the confidence interval of our study. The results suggest a similar trend in both countries although the study in Sweden only included patients with bitewing radiographs (94% of all eligible patients), whereas in our data, more than half of the patients had no radiographs taken. The mean DMFT value in Sweden was 1.92 for 14–year olds and 2.33 for 15-year olds. In our data, the mean DMFT value was 1.62, indicating a slightly better caries situation in Finland.

In Northern Norway, 26% of 18-year-olds had at least one molar with an untreated or previously treated deep carious lesion extending to the inner quarter of dentine [3]. In our sample, 21% of patients with radiographs (10% of all patients) had an untreated or previously treated deep carious lesion to the inner quarter of dentine. The figures are not entirely comparable as the age of our study population was younger than in the Norwegian study. In addition, our study included both premolars and molars and the Norwegian study included only molars. It could be assumed the Norwegian prevalence figures would be higher if premolars had also been included. The mean DMFT values were 5.7 among 18-year-olds in Northern Norway [3] and 4.0 among 17-year-olds in Finland [25]. In conclusion, these results suggest a more severe caries situation in Northern Norway compared to Finland.

The multivariable logistic regression analysis showed an association between a higher DMFT value and the risk of having at least one untreated deep carious lesion to the inner half of dentine. This result is not surprising as previous caries experience has been shown to predict future caries development [26]. The result is also in line with a previous study [3]. The M and F components of the DMFT index reflect past caries experience and even though the distribution of D, M, and F components was not analyzed in our sample, the development of new deep carious lesions in high DMFT individuals indicates that previous caries control measures were not effective.

Assessed high caries risk was associated with an increased risk of having at least one untreated or previously treated deep carious lesion to the inner quarter of dentine. Caries risk assessment, need for caries control measures, and determination of recall interval are mostly based on the clinical assessment by the dentist (Chief Dental Officer of the City of Helsinki, personal communication). The underlying factors behind the assessed high caries risk could have resulted in the development of deep carious lesions but also the presence of untreated and previously treated deep carious lesions could have led to the individual being assessed at high caries risk category. In both scenarios, it seems that high caries risk individuals were recognized. According to the statistical analysis, assessed high caries risk was also associated with more comprehensive caries control measures taken by the dental professionals. However, there is a need to target the caries control efforts more accurately as for one third of high risk individuals none or little caries control measures were registered.

According to the multivariable logistic regression analysis, female gender was associated with an increased risk of having at least one untreated or previously treated deep carious lesion to the inner quarter of dentine. This association has not been shown in previous studies [1, 3]. The cross-sectional study design did not allow us to identify the reasons for the difference between genders. However, DMFT values have previously been shown to be significantly higher among women compared to men [27, 28]. In the global context, there might be cultural and social reasons for higher DMFT values among women. However, this is unlikely in our urban, well-educated study population. Genetic and hormonal factors have also been suggested as possible explanations for higher caries prevalence among women but further studies are needed to clarify their role in the multifactorial caries process [28, 29].

The present international guidelines recommend either selective excavation to soft dentine or stepwise excavation when a carious lesion extends to at least the inner third of dentine [2]. This recommendation is repeated in the Finnish national guidelines [30]. A recent questionnaire study showed 64% of dentists in Finland to prefer either selective or stepwise excavation for a deep carious lesion [16]. However, in our study sample, almost 60% of deep carious lesions extending to at least the inner third of dentine were managed with complete excavation, which is in contrast with the present guidelines and the results of the questionnaire study. Comprehensive set of follow-up radiographs was not available but based on the available radiographic evidence complete excavation was carried out to firm or hard dentine, which is considered overtreatment, jeopardizing the pulpal vitality unnecessarily [2]. If the result is extrapolated to the entire source population, approximately 200 teeth would have been managed with an unnecessary risk of a pulpal exposure in the entire age group. Continuing education is needed to ensure deep carious lesions are diagnosed and managed according to the best scientific evidence.

In conclusion, one-fifth of 14- to 15-year-old adolescents had at least one untreated or previously treated deep carious lesion to the inner half of dentine or deeper. Complete excavation was the most common method chosen for the management of deep carious lesions extending to the inner third of dentine, which is in contrast with the present international and national guidelines.

## Data Availability

The data that support the findings of this study are not publicly available due to confidentiality reasons. The anonymized statistical data is available from the corresponding author (K.C.) upon reasonable request.
