# Correction to: Deep carious lesions and their management among Finnish adolescents: a retrospective radiographic study

**DOI:** 10.1007/s00784-023-05086-z

**Published:** 2023-05-27

**Authors:** Katri Croft, Sari Kervanto-Seppälä, Eero Kerosuo

**Affiliations:** 1 Department of Oral and Maxillofacial Diseases, University of Helsinki, PO Box 41 (Haartmaninkatu 1), 00014 Helsinki, Finland; 2 Vantaa Health Center, Vantaa, Finland

**Correction to: Clinical Oral Investigations**

**https://doi.org/10.1007/s00784-022-04599-3**

In the list of authors, the surnames and first names are in the wrong sequence. The correct forms of author names (first name, surname) are: Katri Croft, Sari Kervanto-Seppälä and Eero Kerosuo.

The original article has been corrected.

